# Interaction of Cultural, Biological, and Varietal Controls for Management of Stalk Borers in Louisiana Sugarcane

**DOI:** 10.3390/insects10090305

**Published:** 2019-09-19

**Authors:** Thomas E. Reagan, Megan M. Mulcahy

**Affiliations:** Department of Entomology, Louisiana State University AgCenter, Baton Rouge, LA 70803, USA

**Keywords:** sugarcane, *Diatraea saccharalis*, integrated pest management, *Eoreuma loftini*, Louisiana, stalk borer

## Abstract

*Diatraea saccharalis* F is considered the most important pest of sugarcane in the United States. This article focuses on the history of pest management as it relates to the control of this stem borer in Louisiana sugarcane, and how control practices have become more in tune with integrated pest management paradigms. Various pest management strategies are employed against *D. saccharalis* and the interactions between each of these provide farmers with the tools needed to curb damaging infestations. However, the invasion of the Mexican rice borer, *Eoreuma loftini* (Dyar), and other confounding environmental factors have presented farmers, consultants, and researchers with new pest management challenges. We address these challenges and provide an overview of ongoing developments, particularly in the Louisiana sugarcane pest management program.

## 1. Introduction

The sugarcane borer, *Diatraea saccharalis* F (Lepidoptera: Crambidae), is native to the West Indies, as well as Central and South America. It was introduced into Louisiana in the 1850s due to the continued importation of sugarcane for production in the state [1]. Since its introduction, *D. saccharalis* has long been regarded as the most damaging insect pest on the crop. Adults of *D. saccharalis* are drab beige in color and have an inverted v-pattern of dots on their wings [2]. Female moths oviposit in clusters on the leaves of sugarcane plants and other grasses. Once the eggs hatch, neonate larvae migrate to and feed within leaf sheaths [3]. After about 10 days, the larvae bore into sugarcane stalks, where they continue to develop through several molts. The larvae then pupate within expanded feeding tunnels inside the sugarcane stalk and eventually emerge as adult moths. In mature plants, damage from the borers cause the tops of the sugarcane to weaken or die, leading to lodging during heavy infestations. In young plants, *D. saccharalis* feeding can kill the leaves within the whorl, resulting in a condition known as dead tops, or “dead heart”. Ultimately, sugar production per hectare is reduced by approximately 0.61% for every 1% of sugarcane internodes bored. Severe infestations can cause yield losses of over 20% in affected fields [4].

This review follows the history of *D. saccharalis* management in Louisiana and the ways in which it has changed to align with new integrated pest management outlooks. The review also looks at how the management of *D. saccharalis* influences the management of other stem-boring pests within the area and how it has affected sugar production in the region.

## 2. A Brief History of Stalk Borer Management in Louisiana

Well before the use of modern pesticides, pest management in the early 1900s emphasized the robust use of common-sense cultural practices for managing this insect in sugarcane. Some recommended cultural control tactics included improvement of field hygiene practices, burning of sugarcane before harvest, manipulation of planting dates, and strategic flooding of fields [5]. One of the most important *D. saccharalis* management tactics at the time was to have all sugarcane growers in Louisiana plant non-insect-infested seed cane [6]. Growers were encouraged to submerge sugarcane in hot water to kill borer larvae prior to planting and to manually remove severely damaged plants during the early growing season [7].

An increase in the availability and affordability of insecticides in the 1940s and early 1950s shifted focus away from cultural control practices, which were typically considered labor intensive [2]. Initially, inorganic cryolite and the botanical ryania were applied to sugarcane to reduce *D. saccharalis* infestations. However, these insecticides provided less than 50% control [8]. As such, they were later replaced, in the mid-to-late 1950s, by organic chlorines or chlorinated hydrocarbons, such as endrin [9]. This class of insecticide was far more effective, with Louisiana Agricultural Experiment Station and USDA-ARS station scientists demonstrating extreme efficiency with both foliar sprays and granules [10]. Unfortunately, the widespread use of these insecticides had unanticipated problems that were felt in sugarcane and other cropping systems. Insect populations were being subjected to repetitive applications of a single class of insecticide, leading to extreme selective pressure [11]. This resulted in the evolution of resistance to pesticides in several pest species. Insecticide resistance against endrin and endosulfan was eventually reported in populations of *D. saccharalis* in Louisiana [12]. In the late 1950s, it also became increasingly apparent that the massive, global use of pesticides was having severe ecological impacts [13,14]. For example, organochlorine pesticides were shown to negatively impact many organisms, including nontarget insects, birds, fish, and snakes, besides numerous other vertebrate and invertebrate species [15].

The concept of integrated pest management (IPM) was developed in response to a mounting record of insecticide failures and to mitigate environmental disasters [14]. Integrated pest management itself can be loosely defined as a decision-making process based on a thorough knowledge of the pest and its tritrophic and environmental interactions [13,16]. It is concerned with using multiple pest management tactics in conjunction with threshold models for the control of insect pests or diseases in agricultural crops [13,16]. The main purpose of IPM is to prevent economically damaging outbreaks of pests and decrease pesticide resistance while also reducing the risks to human health and the environment by reducing the use of chemical insecticides, herbicides, and fungicides on crops [17]. The development of the pest management program against *D. saccharalis* exemplifies the switch from the indiscriminate use of insecticides to the use of integrated control methods. This includes a return to cultural control tactics and greater emphasis being placed on sustainable pest management strategies, such as varietal resistance, biological control, and green chemistries.

## 3. Host Plant Resistance in Sugarcane Cultivars

Recognition of possible stalk borer resistance in sugarcane was cited as early as 1902 by Stubbs and Morgan [18]. The development of sugarcane varieties, with improved host plant resistance against the sugarcane stalk borer, undoubtedly occurred prior to the development of modern insecticides. However, the mechanisms of resistance were poorly understood [2]. Studies by Coburn and Hensley (1972) [19] compared larval survival and establishment of *D. saccharalis* on resistant (NCO 310) and susceptible (CP 44-101) cultivars. In this study, appression of the sugarcane leaf sheath was shown to have an effect on the boring behavior of the sugarcane borer. In the resistant cultivar (NCO 310), natural boring by *D. saccharalis* occurred in the fifth leaf sheath down from the whorl. In contrast to this, boring in the susceptible cultivar (CP 44-101) typically occurred in the top first or second leaf sheath, which were open (not appressed). The benefits of leaf sheath appression were tested by physically tying leaf sheaths of CP 44-101 closed while simultaneously prying the upper sheaths of NCO 310 open [19]. They found reverse mortality, with more survival in NCO 310 and less in the manually “closed” CP 44-101. Other applied entomologists have cited various characteristics in sugarcane which are correlated to borer resistance in the crop, including narrow leaves, high fiber content, light stalk color, heavy wax coating, leaf shedding, thin stalks, longleaf spindles, erect leaves, high vigor, juice content, low leaf senescence, and attractiveness to egg-laying moths [20,21,22].

Additionally, early research conducted by Mathies and Charpentier (1969) [23] suggested that rind hardness may confer resistance to sugarcane borers due to its negative effects on the survival of neonate larva. This was followed up by studies that confirmed the benefits of rind hardness in reducing the prevalence of *D. saccharalis* infestations with the use of a durometer (penetrometer) [24]. These results were expanded upon in Reagan and Martin (1982) [25]. They were able to assess eight common cultivars at the time, as well as nine experimental station cultivars, dividing the level of resistance into four groupings. This included the traditional method of evaluating cultivar resistance using percentage of bored internodes, in addition to determining the distinct number of adult emergence holes [26]. These measures are still used as an assessment tool today to monitor *D. saccharalis* injury and infestation levels. Rind hardness and fiber content remain important tools in the development of resistant cultivars to combat *D. saccharalis* [27].

In addition to extensive work conducted against *D. saccharalis* [20], studies looking at resistance to the invasive borer *Eoreuma loftini* (Dyar) (Lepidoptera: Crambidae) were initiated in 2001 [28] and continued by Wilson (2011) [21] and Wilson et al. (2015) [29]. *E. loftini* (the Mexican rice borer) is another stalk borer invading sugarcane in Louisiana. Its behavior and habits are very similar to those of *Chilo saccariphagus* Bojer (Lepidoptera: Crambidae) and *Eldana saccharina* Walker (Lepidoptera: Pyralidae) [30,31]. Mechanisms used to manage these pests have been well researched in Southern Africa and may be incorporated into the Louisiana sugarcane pest management strategy through collaboration in the future. For instance, the South African cultivars N21 and N24 were found to be resistant to both *E. saccharina* and *E. loftini*, whereas N27 was susceptible [22]. Of the South African cultivars, N21 has excellent drought tolerance [32]. Evidence demonstrates that, like *E. saccharina*, the Mexican rice borer responds favorably to water stress in sugarcane plants [31]. Drought promotes outbreaks of phytophagous insects due to elevated plant nutrient levels (especially nitrogen), reductions in the plants ability to defend itself, and lastly, through the creation of improved temperature niches in the physical environment, allowing for increases in survivability and developmental rate [33,34,35]. Thus, tolerance to water stress within sugarcane varieties can be used successfully within sugarcane cultivars to manage stem borer populations during adverse climatic conditions. Resistant cultivars, such as N21 and N24, also hinder stalk penetration by borer larvae, which are therefore more exposed to predation, parasitism, and insecticide applications (since they are largely protected once inside the stalk) [32]. This is particularly true of *E. loftini* larvae, which typically pack their feeding tunnels tightly with excrement, thus isolating them from external control measures [36]. Reduced penetration as a means of host plant resistance can therefore be used in conjunction with IPM tools such as biocontrol and judicious insecticide use to control stem-boring pests in sugarcane [37]. The tunnels of *D. saccharalis* are wider and more open, thus allowing improved management through arthropod predation and parasitoids [38]. However, this pest can still be difficult to control once inside the stalk. Research is currently assessing the resistance of various cultivars, both local and foreign, to identify unique resistant germplasms for the development of commercial cultivars that are able to reduce infestations of both *D. saccharalis* and *E. loftini* pest populations in Louisiana [22].

Furthermore, annual assessments of stalk borers (*D. saccharalis* and *E. loftini*) in four or five replication experiments have been conducted over a 10–12-year period at the LSU AgCenter and the USDA-ARS at Houma, Louisiana, United States to evaluate new cultivars. These are similar to those conducted by Reagan and Martin (1982) [25]. Each annual program starts out with at least 100 seed lines [22,39]. In addition to the measurements previously mentioned for monitoring stem borer infestations, a sequential plan for the sugarcane borer was developed for minimizing and streamlining sampling time in the field [40]. Currently, the following varieties are available and recommended to growers for resistance to stem borers in Louisiana: L 99-226, L 01-299, and HoCP 04-838 [41]. Of the recommended varieties for the 2019 growing season in Louisiana, L 01-283 and Ho 12-615 are considered mildly resistant to the sugarcane borer. A further five of the recommended commercial varieties (HoCP 96-540, HoCP 00-950, HoCP 09-805, L 11-183, and L 12-201) are susceptible to damage from *D. saccharalis* [41].

## 4. Development of Sustainable Insecticides for Use in Louisiana Sugarcane

Organophosphates (acetylcholinesterase inhibitors), such as azinphos-methyl and monocrotophos, replaced the organochloride (GABA-gated channel blocker) endrin after that insecticide was banned. However, monocrotophos had problems with resistance in the Lower Rio Grande Valley [42,43]. After suspension and restriction of azinphos-methyl (loss of label in 1995 for fish kills), these insecticide control methods were finally replaced by the insect growth regulator tebufenozide [42,44]. The first ever evaluation for *D. saccharalis* control by tebufenozide occurred in 1993 [45]. Now, more than 80% of all the tebufenozide insecticide produced in the United States is applied to Louisiana sugarcane annually. This insecticide is considered to be narrow range and minimum risk [40,46]. It is a molting accelerator compound which affects larval growth in some lepidopterous insects, particularly those in the families Pyralidae and Crambidae [46]. Unlike most insecticides, which rapidly kill insects a few hours after exposure, tebufenozide acts only during the molt, when insects shed their exterior cuticle. Larvae exposed soon after the molt show no chemical effects for several days until after the next molt [47,48]. In addition, tebufenozide was one of the few insecticides developed at the time which did not suppress nontarget crickets, beneficial spiders, and other selected arthropods [49]. This is in contrast to the insecticide lambda-cyhalothrin, which is a broad-spectrum pyrethroid (sodium channel modulator) that is effective against *D. saccharalis* and several nontargeted secondary pests [42], including the West Indian canefly, *Saccharosydne saccharivora* Westwood (Hemiptera: Fulgoridae), and yellow sugarcane aphid, *Sipha flava* Forbes (Hemiptera: Aphididae). Although this made pyrethroid insecticides, such as lambda-cyhalothrin, a useful tool for farmers, they have since begun to be phased out due to their negative impacts on beneficial arthropods in the agroecosystem, pest resurgence (especially in aphid populations), and insecticide resistance problems [50,51].

In sugarcane biological control studies, stem borer parasitoids that were exposed to sugarcane leaves from insecticide-treated field plots showed very low levels of mortality to tebufenozide [49]. As such, tebufenozide use in sugarcane allowed for increased predation and parasitism of *D. saccharalis* to such an extent that insecticide applications on sugarcane were reduced from three applications a year to just half an application per year. This resulted in tebufenozide receiving the presidential Green Chemistry Challenge Award for insecticide discovery research in 1998 from the president of the United States and the Federal Environmental Protection Agency [46].

Other research programs have also assessed the potential for using different economic injury thresholds for managing *D. saccharalis* with insecticides, together with susceptible and resistant commercial cultivars. Posey et al. (2006) [39] conducted a two-year study to assess this integrated approach. In this four-replication test, susceptible cultivars (LCP 85-384 and Ho CP 91-555) exceeded 40% bored internodes in untreated plots. However, when treated with tebufenozide, damage levels in susceptible cultivars did not differ significantly from the levels attained in resistant cultivars. Furthermore, a higher threshold level could be used (10% bored internodes vs. 5% bored internodes) with tebufenozide, without compromising the level of control.

Due to its efficacy, Louisiana experienced widespread use of tebufenozide, which represented 90% of all the foliar applications of insecticides in sugarcane during the 2007 growing season alone [52]. This increased concerns regarding the potential for the development of resistance against the chemistry in populations of *D. saccharalis*, which has been an on-going problem in Louisiana sugarcane pest management [51]. In 2008, researchers selected for resistance to tebufenozide in laboratory-reared *D. saccharalis*. They obtained a 27.1-fold increase in LC50 after 12 generations of the pest were subjected to selection with tebufenozide [53]. Thus, insecticide resistance management strategies were highlighted as being necessary to preserve *D. saccharalis* control tactics for the Louisiana sugarcane industry [51].

Fortunately, success with tebufenozide paved the way for the development and introduction of other selective, sustainable insecticide chemistries. Recent studies have demonstrated the effectiveness of a chitin inhibitor (novaluron) for *D. saccharalis* control [38]. Like tebufenozide, novaluron has no measurable impact on nontarget arthropods and can be used in conjunction with biocontrol strategies. Two diamide insecticides, namely, chlorantraniliprole and flubendiamide, are also extremely effective for the management of lepidopteran stem borers [38,54]. These insecticides attack insect ryanodine receptors and are active against other lepidopteran pests too [54,55]. Fortunately, their high-level selectivity means that nontarget organisms are relatively safe if they are exposed to the diamides [56]. While these insecticides are labeled for use in Louisiana sugarcane, studies comparing these insecticides with industry standards for *D. saccharalis* and *E. loftini* are ongoing [38]. However, the effectiveness of these insecticides, together with on-going tebufenozide use, have the potential to provide farmers with multiple pest management options to better control stem borer infestations and to curb the likelihood of insecticide resistance through reduced selective pressure.

Scouting by farmers and other stakeholders is an important tool that can improve the application of insecticides in a manner that promotes IPM for the control of *D. saccharalis* in Lousiana. Farmers, extension officers, and consultants scout for *D. saccharalis* from mid-June to September by looking for larval feeding scars on leaf sheaths and by looking for larvae, as well as entrance and exit holes near internodes and along the stalk [57]. Effective monitoring of this pest allows farmers to spray insecticides on sugarcane only when the borers are present at damaging levels. The economic injury level for *D. saccharalis* is 6–12% internodes bored (depending on the variety selected), at which point action should be taken and insecticides applied [4]. This avoids unnecessary chemical applications and can reduce costs, environmental contamination, and insecticide resistance. However, scouting for *D. saccharalis* is time consuming, laborious, and by the time farmers can see the effects of stem borer damage, it is too late to treat fields [40]. Although alternative scouting methods, such as black light and pheromone traps, have been assessed in the past [58], correlating trap catches with larval infestations and crop damage is difficult for many stem borer species [59]. Therefore, Louisiana is reliant on manual scouting techniques for *D. saccharalis* [60]. Due to differences in mating behavior and response to trap design, there has been some success using pheromone traps to monitor *E. loftini* populations. Capture of 20 moths per trap per week roughly corresponds to the action threshold for this species in sugarcane [60]. The use of pheromone traps has also been useful in detecting populations of *E. loftini* and for monitoring its invasion into new sugarcane growing regions [38]. Therefore, the efficacy of pheromone traps for stem borer scouting should continue to be researched and improved upon, as well as the use of moth sex pheromones for mating disruption [61].

## 5. The Role of Biological Control and Adverse Weather Conditions in Borer Management

The movement towards more environmentally aware pest management practices can be seen relatively early on in the southeastern United States, with a shift in paradigms regarding the Federal and State USDA-APHIS red imported fire ant, *Solenopsis invicta* Buren (Hymenoptera: Formicidae), eradication program [62]. When interviewed by Rachel Carson (1962) [63], the Head of the Louisiana State University Entomology Department Dr. L.D. Newsom stated that “the imported fire ant eradication program had thus far been a failure”. He went on to say that the infestation had increased in Louisiana since the program was initiated. The failures of such programs eventually led to an abandonment of eradication as a pest management solution, with focus shifting to more localized control tactics [64]. In the case of *S. invicta*, the cancellation of the EPA’s mirex eradication program revealed that in many agricultural areas, the fire ant eradication program had negative effects on crop yields [64,65,66]. Not only did mirex decrease populations of beneficial natural predators, such as spiders, carabids, and staphylinids, but *S. invicta* were also shown to have a positive impact on crop production through predation of *D. saccharalis* larvae [65,66,67]. Additionally, tick populations decreased in areas where imported *S. invicta* had just begun invading northwest Louisiana. This led to reductions in tularemia (rabbit fever) and other tick-borne diseases in human populations due to *S. invicta* predation [68]. This highlighted the benefits of *S. invicta* in Louisiana and the benefits of preserving natural predator and parasitoid populations within sugarcane fields.

Depending on the particular borer attacking sugarcane, biological control via predation has a substantial impact [69]. Although biological control using indigenous and exotic parasitoid wasps and flies has had some success in Florida and the Lower Rio Grande Valley in Texas [43,70], it has been less effective in other regions of the United States [71]. Predation on parasitoids in Louisiana can be severe, thereby reducing the ability of parasitoids to establish and maintain pressure on sugarcane borer populations [72]. The more temperate climate in Louisiana and parasitoid inability to locate hosts in young sugarcane are likely affecting the success of parasitoids such as *Cotesia flavipes* Cameron (Hymenoptera: Braconidae) [73]. Entomopathogens, such as *Beauveria bassiana* Balsamo (Hypocreales: Cordycipitaceae), are unable to curb *D. saccharalis* populations under field conditions [74,75], and although *Bacillus thuringiensis* Berliner reportedly decreases *D. saccharalis* damage by up to 75%, it has yet to be adopted for commercial production [31,76]. Biological control of *E. loftini* is even more problematic due to its cryptic behavior inside the sugarcane stalk [77].

Another hurdle for successful biological control in Louisiana is the prevalence of heavy rains, tropical storms, and other adverse weather conditions. A 4-treatment, 12-replication large-area study was conducted in numerous places within St. Mary, Iberia, and Vermilion parishes in southcentral Louisiana to assess the impact of Hurricane Rita on biological control [78]. This storm surge affected large areas of the state, including sugarcane production regions. Both flooded and nonflooded areas were tested for efficacy of natural biological control in plant and ratoon commercial sugarcane [78]. The study showed that sugarcane in flooded areas experienced a rise in yield losses of between US$1.9 million and US$2.6 million due to increases in pest infestations after the hurricane. The primary entomological role of the hurricane was its impact on biological control, mainly through a reduction in arthropod predation. A significant 2.8-fold reduction in numbers of the predators was observed in impacted fields. Even with a significant 2.4-fold increase in the average number of insecticide applications (for *D. saccharalis* management) in formerly flooded fields, sugarcane growers still incurred substantially higher losses as a result of pest injury [78]. The effects of adverse weather on agriculture and pest populations is of growing concern and should continue to be monitored, as the likelihood of extreme weather events in the region is expected to become more frequent due to warmer waters in the Gulf of Mexico and similar issues related to ongoing global climate change [79,80]. This is especially true since climate change alone is expected to improve conditions for insect pests, thereby increasing the risk of pest outbreaks in sugarcane cropping systems [81].

## 6. Recommended Cultural Control Practices

In the past, a large number of cultural controls were recommended to farmers for the management of *D. saccharalis* in Louisiana sugarcane [82]. Some of these cultural control practices have since been discontinued due to a lack of labor and higher input costs [3]. These include hot water treatments of seed cane, removal of postharvest trash, and targeted manual destruction of damaged plants in the spring [3]. Another formerly common cultural control method was the preharvest burning of sugarcane, which was used throughout the world as a means of reducing in-field pest populations [83]. Preharvest burning had the added benefit of removing extraneous leaf materials, making the sugarcane easier to cut and process [31,83]. However, postharvest sucrose deterioration in sugarcane stalks, loss of soil organic matter, and the negative environmental and health effects caused by sugarcane burning emissions have resulted in pressure on farmers to adopt “green cane harvesting” [84,85]. Thus, preharvest burning can no longer be relied upon to reduce pest numbers. Fortunately, some studies have demonstrated the benefits of unburnt residues and trash blankets on stem borer predators, such as *S. invicta* [84,86].

Current cultural control practices that are used in Louisiana to manage *D. saccharalis* populations include (1) planting noninfested seed cane; (2) reducing overwintering larvae by plowing stubble postharvest; and (3) maintaining crop residues, trash, and broken stalks in fields over winter, so that remaining larvae are killed by low temperatures [57]. Furthermore, maize (*Zea mays* L. (Poales: Poaceae)) acts as a suitable host plant for *D. saccharalis* [40,87]. Therefore, farmers are advised to plant susceptible maize cultivars as far from sugarcane as possible to decrease midsummer migrations of the moth from senescing cornfields [57]. Finally, planting date may also have an effect on *D. saccharalis* populations in sugarcane, with early planted sugarcane being susceptible to increased infestations [88]. Planting date manipulation may also be a useful tool for managing *E. loftini* infestations, however management of this borer would likely improve with early planting [71]. This pest responds well to water-stressed plants, high nutrient content, and availability of cryptic oviposition sites. Therefore, improved water management, judicious nitrogen application, and removal of sugarcane trash are some cultural methods that can be used against *E. loftini* [31]. Of course, this may clash with recommendations to retain plant residues and trash in order to curb *D. saccharalis* numbers. This highlights the need to consider pest interactions in the sugarcane agroecosystem and to manage both stem borer species as a pest complex.

## 7. Conclusions

In conclusion, it is evident that *D. saccharalis* has had a great impact on sugarcane research and production techniques in the region for many years. The Louisiana sugarcane industry exemplifies the move towards more sustainable, economically viable, and environmentally sound farming practices. The evolution of *D. saccharalis* management techniques underscores the trend towards integrated pest management, with the focus shifting from the prescripted use of insecticides to a more holistic system that includes the use of biological control and varietal management. This has the added benefit of reducing input costs and conserving important ecological processes that will continue to aid in the management of this pest. Farmers now have a diverse set of tools with which to control *D. saccharalis*. The invasion of *E. loftini* has presented new challenges to the Louisiana sugarcane industry; however, novel research avenues and management practices are being pursued and developed to address this challenge in a way that enhances and complements the existing insect pest management program in sugarcane.
